# Circular RNA SET domain protein 3 promotes nasopharyngeal carcinoma proliferation, cisplatin resistance, and protein kinase B / mammalian target of rapamycin pathway activation by modulating microRNA-147a expression

**DOI:** 10.1080/21655979.2022.2036907

**Published:** 2022-02-23

**Authors:** Gang Deng, Fei Wang, YiSa Song

**Affiliations:** a Department of Otorhinolaryngology, Wuhan No. 1 Hospital of Hubei Province, Wuhan City, HuBei Province, China; b Department of Otorhinolaryngology, People’s Hospital of Qinghai Province, Xining City, QingHai Province, China

**Keywords:** CircSETD3, microRNA-147a, nasopharyngeal carcinoma, cisplatin resistance

## Abstract

Circular RNA (circRNA) plays a crucial role in the establishment and progression of nasopharyngeal carcinoma (NPC). Understanding the role of circRNA in NPC is helpful to find new therapeutic targets for NPC. The purpose of this study was to explore the effects of circRNA SET domain protein 3 (circSETD3) on protein kinase B (Akt)/ mammalian target of rapamycin (mTOR) signaling pathway and cisplatin (DDP) resistance to NPC and explore its downstream mechanism. The results showed that circSETD3 was upregulated in NPC tissues and was related to DDP resistance to NPC. Functional experiments revealed that circSETD3 knockdown inhibited NPC proliferation and increased DDP sensitivity and apoptosis rate. The promotion effect of circSETD3 overexpression on NPC proliferation and DDP resistance and inhibition effect on apoptosis was reversed by elevated miR-147a. CircSETD3 knockdown or miR-147a overexpression prevented Akt/mTOR pathway’s activation. In terms of the mechanism, circSETD3 acted as a sponge for miR-147a. Xenotransplantation experiments showed that knockdown circSETD3 or DDP treatment could restrain tumor growth, and the effect of DDP was enhanced by knockdown of circSETD3. In conclusion, the results of this study confirm that circSETD3 promotes NPC proliferation and DDP resistance by regulating miR-147a, and circSETD3/miR-147a axis may serve as a potential therapeutic target for NPC in the future.

## Graphical abstract

**Figure:**
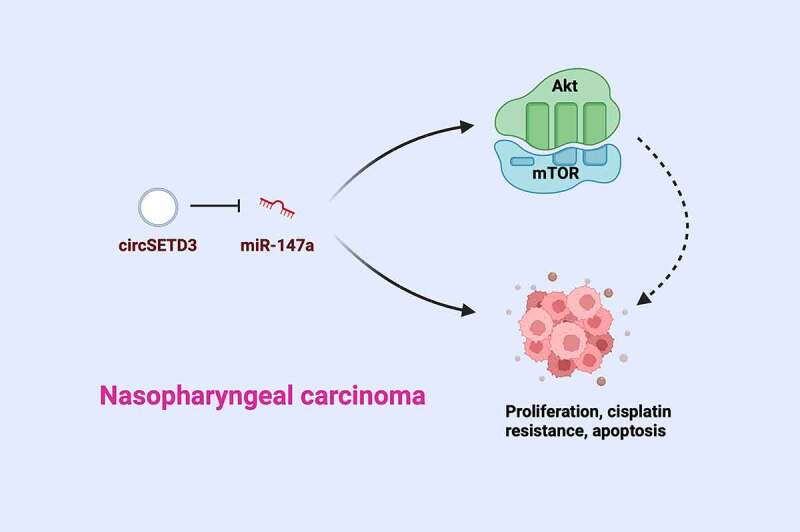

## 1. Introduction

Nasopharyngeal carcinoma (NPC), a highly malignant nasopharyngeal mucosal epithelial tumor, is the most common head and neck tumor [1]. NPC mainly presents with rhinitis swelling, neck lymph node enlargement, and intracranial nerve injury, and also produces spindle cells with epithelial-mesenchymal transition and CSCs characteristics [2,3]. The conventional treatment approaches for NPC include radiotherapy, surgery, or chemotherapy. However, surgical approaches have led to unpleasant outcomes [4,5]. Although radiation or chemotherapy have been the most widely used modalities against local tumor metastasis, NPC resistance to radiotherapy and chemotherapy remains a major clinical challenge. The total 5 years survival rate among advanced NPC patients is only 65% [6]. Hence, understanding the underlying molecular mechanisms leading to NPC resistance is urgently needed to help in handling NPC resistance and develop better therapeutic approaches.

Circular RNA (circRNA) is a stable and conservative special RNA with a covalent closed-loop structure, and widely exists in different tissues and organs with varying levels of expression, and broadly participates in the occurrence and development of various cancers in different ways [7–11]. As a member of the circRNA family, CircSETD3 has been reported as a novel proto-oncogene that accelerates resistance to Gefitinib in non-small cell lung cancer [12,13]. Additionally, circSETD3 has been differentially expressed in gastrointestinal malignancies [14]. A recent study has demonstrated the elevation of circSETD3 and the promotion of invasion and migration in NPC [15]. However, the role of circSETD3 in NPC proliferation, apoptosis, and mechanisms of resistance to cisplatin (DDP) has never been investigated.

The protein kinase B (Akt)/ mammalian target of the rapamycin (mTOR) pathway is crucial in cancer malignancy. The binding of PTEN to growth factors such as EGFR changes the structure of Akt and leads to its activation, which regulates the proliferation, apoptosis, and migration of cancer cells by activating downstream substrates such as Bax and caspase-3 through phosphorylation [16,17]. The blocking of inactivated AKT/mTOR pathway on the malignant behavior of NPC has been reported by various studies [18–20]. However, the possible effects of circSETD3 on the AKT/mTOR pathway in NPC remain unclear.

In this study, circSETD3 in NPC and resistant cells was changed via transfection and DDP induction in NPC DDP drug-resistant cells, informing that circSETD3 facilitates NPC proliferation and DDP resistance but represses apoptosis via activation of AKT/mTOR pathway and being as a competing endogenous RNA (ceRNA) of miR-147a.

## 2. Materials and methods

### 2.1 Clinical samples

Samples were collected from Wuhan No. 1 Hospital of Hubei Province from March 2013 to August 2017. A total of 78 NPC cases (including 32 DDP resistant and 46 DDP sensitive cases) and para-carcinoma normal tissues (distance from NPC tissue≥5 cm) were used in the study. All patients received nasopharyngeal tumor biopsies and were diagnosed with primary NPC based on pathological examination. After rapid freezing in liquid nitrogen, all tissue samples were stored at −80°C. This study was approved by the Ethics Committee of Wuhan No. 1 Hospital of Hubei Province, and written consent was gained from all patients (approval number: W201211685h

### 2.2 Cell culture

Four NPC cell lines (SUNE1, CNE2, HNE1, and C666) and immortalized nasopharyngeal epithelial cell lines (NP69) (American Type Culture Collection, US) were cultured in Roswell Park Memorial Institute-1640 (RPMI) medium (Invitrogen, Carlsbad, CA, USA) supplemented with 10% fetal bovine serum (100 U/mL) and penicillin-streptomycin (100 U/mL). For acquisition of DDP resistant cell lines, the parental cell line SUNE1 was treated with 0.5 µg/mL DDP (QILU PHARMACEUTICAL) for 3 weeks. The cells were then cultured with elevated concentrations of DDP (2, 4, 6, 8, 10 µg/mL) for 21 weeks. The SUNE1 cells were finally treated with 1 µg/mL DDP to maintain DDP resistance (SUNE1/DDP). Cell counting kit (CCK)-8 was used to determine the 50% inhibitory concentration (IC_50_ value) to analyze the resistance of SUNE1 cells to DDP.

### 2.3 Cell transfection

Small interfering RNA targeting circSETD3 (si-circSETD3) and negative control siRNA (si-NC), miR-147a mimic and mimic NC were synthesized commercially by GenePharma (Shanghai, China). For overexpressing circSETD3, the full-length circSETD3 sequence was inserted into the pcDNA3.1 vector (Shanghai Gene Medicine Co., Ltd.) as described elsewhere [21]. The transfection of the above reagents into SUNE1/DDP cells was carried out with Lipofectamine 2000 (Thermo Fisher Scientific). The transfected cells were then collected for subsequent assays.

### 2.3 The detection of IC_50_ via CCK-8

SUNE1 cells were seeded into 96-well plates and treated with DDP (0, 2, 4, 8, 16, and 32 μg/mL) for 48 h. The cell viability was determined by the CCK-8 Assay kit (Dojindo Laboratories, Tokyo, Japan) according to the manufacturer’s instructions. The absorbance values were measured at 450 nm via a SpectraMax 250 analytical instrument (Molecular Equipment, Inc. US).

### 2.4 Colony formation assay

The cell proliferation was detected via colony formation assay [22]. SUNE1/DDP cells in the logarithmic growth phase were collected and seeded in 6-well plates at a density of 500–1000 cells per well and cultured in a complete medium supplemented with 4 μg/mL DDP and 30% Fetal bovine serum (FBS). After 14 d, the plate with the cells was removed from the incubator. The medium was discarded, and the cells were fixed in 4% paraformaldehyde, stained in 0.2% crystal violet (Sigma-Aldrich; Merck KGaA), dried, and photographed with a light microscope. Colonies of over 50 cells were counted. The experiment was repeated three times.

### 2.5 Flow cytometry

Flow cytometry was used to investigate apoptosis as previously described [23]. Approximately 10^4^ SUNE1/DDP cells were seeded in 6 well plates. DDP (4 μg/mL)was added to each well. The cells were then harvested, detached with 0.25% trypsin, centrifuged at 2500 rpm, and the supernatant was discarded. The precipitate was transferred to a 1.5 mL Eppendorf tube and resuspended in 1 mL phosphate buffer saline (PBS). The cell suspension (100 μL) was incubated in tubes containing 5 μL fluorescently labeled annexin V/fluoresceinisothiocyanate (FITC) and 10 μL propidium iodide (20 μg/mL). Later, the mixture was mixed with 400 μ L staining buffer and then loaded in the flow cytometer (BD Biosciences, USA) for analysis. For each test, the cells were loaded, and the data were analyzed via Cell Quest software (BD Biosciences)

### 2.6 Reverse transcription-quantitative polymerase chain reaction (RT-qPCR)

The RT-qPCR was done as previously described [24]. In summary, total RNA was extracted from tissues and cell lines using TRIzol reagent (Ambion, Life Technologies, USA). The cDNA was reverse transcribed using the TaqMan miR Reverse Transcriptase Kit (Applied Biosystems-Life Technologies), and mRNA or circRNA’s cDNA were synthesized with the ImProm-II Reverse Transcription System (Promega). SYBR Premix EX Taq™ (Takara) was used for qPCR analysis. The relative gene expression was calculated through 2^−ΔΔCT^. The relative expression of the genes was normalized to U6 and β-actin. Primer sequences used are shown in Table 1.

**Table 1.** RT-qPCR primer sequences

	Primer sequences (5’– 3’)
β-actin	Forward: 5’- ATAGCACAGCCTGGATAGCAACGTAC-3’
	Reverse: 5’- CACCTTCTACAATGAGCTGCGTGTG-3’
U6	Forward: 5’- ATTGGAACGATACAGAGAAGATT-3’
	Reverse: 5’- GGAACGCTTCACGAATTTG-3’
CircSETD3	Forward: 5’- TTCGGTATCTTCAGTCCACACA-3’
	Reverse: 5’- TTCCTTTGGTGACACAGTTGC −3’
MiR-147a	Forward: 5’- CCCCTATCACGATTAGCATTAA-3’
	Reverse: 5’- CCCAAGCTTTTATGTGGTTGTT −3’

### 2.7 Western blot

The total proteins were extracted from cells and tissues using Radio-Immunoprecipitation assay (RIPA) buffer (Beyotime Institute of Biotechnology). The protein concentrations were determined using bicinchoninic acid Protein Assay Kit (Beyotime Institute of Biotechnology). The protein separation (10 µg) was done using 10% sulfate-polyacrylamide gel electrophoresis and electroblotting, and the proteins were transferred onto the Polyvinylidene fluoride membrane (EMD Millipore). Later, the membrane was blocked using 5% skimmed milk in PBST and incubated with the following primary antibodies: glyceraldehyde-3-phosphate dehydrogenase (GAPDH, ab8245, Abcam, 1:1000), cleaved caspase-3 (ab2302, Abcam, 1:1000), Bax (ab32503, Abcam, 1:1000), Bcl-2 (12789-1-AP, Proteintech, 1:1000), Akt (9272, Cell Signaling Technology, 1:1000), p-Akt (9271, Cell Signaling Technology, 1:1000), mTOR (2972, Cell Signaling Technology, 1:1000), p-mTOR (2971, Cell Signaling Technology, 1:1000), and horseradish peroxidase-conjugated secondary antibody (ab6721, Abcam). Visualization of the protein bands was done using an enhanced chemiluminescence reagent (Beyotime Institute of Biotechnology), and the protein expression was quantified via Image Lab software (version 4.1; Bio-Rad Laboratories, Inc.). β-actin was used as an internal reference.

### 2.8 The dual luciferase assay

The pmirGLO-circSETD3-wild type (WT)/mutant type (MUT) reporting plasmid was obtained from Shanghai Genentech Pharmaceutical Co., Ltd. The co-transfection of 0.6 µg circSETD3-WT/MUT and 100 nM mimic NC or miR-147a mimic into SUNE-1 cells was done using Lipofectamine® 2000 according to the manufacturer’s instructions. Luciferase activity was assessed through the dual-luciferase reporter assay system (Promega Corporation). The relative luciferase activity was normalized with Renilla Luciferase activity.

### 2.9 RNA immunoprecipitation analysis (RIP)

The lysis of SUNE-1 cells was done using the EZMagna RIP kit (Millipore, USA) as per the manufacturer’s protocol and then incubated with RIPA buffer containing magnetic beads conjugated with human anti-Argonaute2 (Ago2) antibody (Millipore). Normal mouse Immunoglobulin G (IgG) (Millipore) was used as an NC. There was the incubation of the sample with protease K, the immunoprecipitated RNA extraction, and RT-qPCR analysis of the purified RNA.

### 2.10 Xenograft

The animal experiments were authorized by the Animal Ethics Committee of Wuhan No. 1 Hospital of Hubei Province. Thirty-six male BALB/c nude mice (Shanghai Laboratory Animal Center, Chinese Academy of Sciences), aged from 6 to 8 weeks, were assigned into 6 groups: the control, the DDP, the si-NC, the si-circSETD3, the DDP + si-NC, and the DDP + si-SETD3 (n = 6). After 1-week of adaptive feeding, the SUNE1 cells in the logarithmic phase were harvested the subcutaneously injected (0.2 mL) into nude mice at a density of 10^7^ cells/mL. When the tumor reached about 300 mm^3^, the DDP was intraperitoneally injected (25 mg/kg, twice a week) in DDP treated group, with a corresponding volume of normal saline in the rest group for 5 weeks. The weekly measurement of the long and short diameters of the tumors was done with vernier calipers. At the end of the experiment, the mice were anesthetized, and the tumor was removed to measure the weight. The volume calculation was done according to the formula: volume = (length/width ^2^)/2.

### 2.11 Immunohistochemistry

Xenografted tumor specimens from nude mice were fixed in 4% paraformaldehyde and then embedded in paraffin. After dewaxing, hydration, antigen retrieval, and sealing, 5 μm sections were incubated overnight with specific primary antibodies at 4°C. Primary antibodies were as follows: Ki67 (ab15580, Abcam, 1: 1000), cleaved caspase-3 (ab2302, Abcam, 1: 1000), Bax (ab32503, Abcam, 1: 1000), Bcl-2 (12789-1-AP, Proteintech, 1: 1000). The sections were then incubated at room temperature for 30 min with anti-mouse/rabbit secondary antibody (Abcam, USA). Diaminobenzidine (DAB) kit (Sigma, USA) was used for staining. Images were obtained under a microscope with appropriate magnification (Olympus, Japan).

### 2.12 Data analysis

The data were analyzed using IBM SPSS Statistics 20.0 software (IBM, USA). The data were presented as mean ± standard deviation. The differences between the two groups were assessed using a t-test, while the differences between groups were analyzed through one-way analysis of variance (ANOVA) and Tukey’s post hoc test. *P* < 0.05 was considered statistically significant.

## 3 Results

### 3.1 Elevated circSETD3 in NPC is connected with DDP resistance

**Figure 1.**
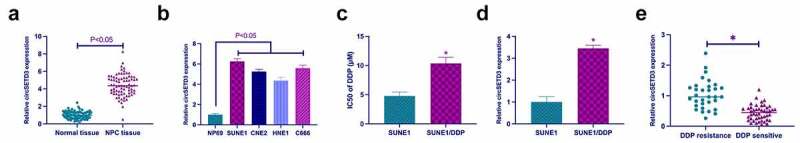
Up-regulated circSETD3 in NPC is implicated with DDP resistance. (a) RT-qPCR detection of circSETD3 in NPC and para-cancerous normal tissues; (b) RT-qPCR detection of circSETD3 in NPC cell lines (SUNE1, CNE2, HNE1, and C666) and immortalized nasopharyngeal epithelial cell line (NP69); (c) CCK-8 detection of IC_50_ values of SUNE1 and SUNE1/DDP cells; (d) RT-qPCR detection of circSETD3 in SUNE1 and SUNE1/DDP cells; (e) RT-qPCR to detect the expression of circSETD3 in DDP resistant and DDP sensitive patients. The data were presented as mean ± standard deviation (n = 3); vs the SUNE1 group, *P < 0.05.

To investigate the role of circSETD3 in NPC, the expression of circSETD3 in NPC was assessed by RT-qPCR. The results confirmed significantly elevated circSETD3 in NPC tissues and cells (Figure 1(a,b)). Analysis of clinicopathological features confirmed a close connection of upregulated circSETD3 and TNM stage and metastasis of NPC patients (Table 2). Subsequently, DDP-resistant SUNE1 cells were obtained by treatment with different concentrations of DDP. The CCK-8 results confirmed an increased IC_50_ value in SUNE1/DDP cells compared to SUNE1 cells (Figure 1(c)). Then, the effect of DDP treatment on circSETD3 expression was examined in SUNE1 cells (Figure 1(d)). According to the observations, circSETD3 expression was significantly elevated in SUNE1/DDP cells compared to parental SUNE1 cells. In addition, the expression of circSETD3 in DDP sensitive patients was significantly down-regulated compared to the DDP resistant patients (Figure 1(e)).

**Table 2.** Upregulated circSETD3 is closely associated with TNM stage and metastasis of NPC patients

Features	Groups	n	CircSETD3 expression		*P*
			High expression group (n = 39)	Low expression group (n = 39)	
Age (years)	< 55	27	11	16	0.2340
	55 or more	51	28	23	
Gender	Female	42	24	18	0.1730
	Male	36	15	21	
Tumor size (cm)	< 5	32	13	19	0.1672
	5 or more	46	26	20	
Lymphatic metastasis	Yes	53	31	22	0.0290
	No	25	8	17	
TNM staging	I + II	48	19	29	0.0199
	III + IV	30	20	10

### 3.2 Knockdown circSETD3 inhibits NPC proliferation and DDP resistance

**Figure 2.**
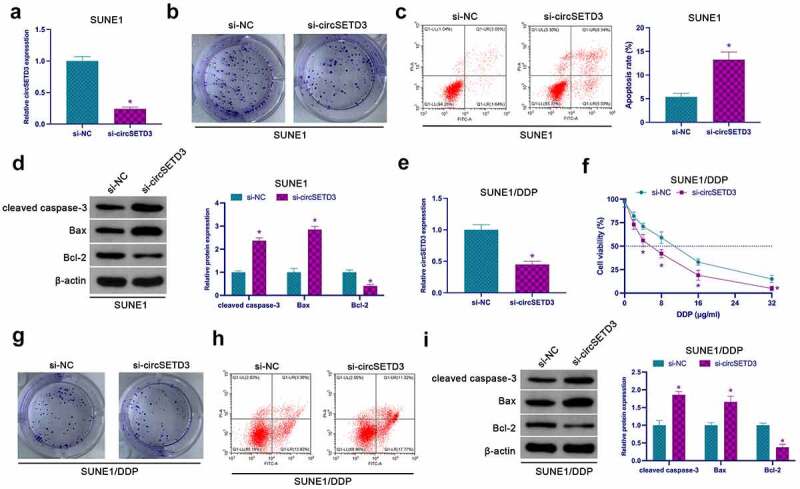
Repressive circSETD3 restrains NPC proliferation and DDP resistance. (a) RT-qPCR detection of circSETD3 in SUNE1 cells transfected with si-circSETD3; (b) Colony formation assay for detection of the proliferation of SUNE1 cells transfected with si-circSETD3; (c) Flow cytometry detection of apoptosis of SUNE1 cells transfected with si-circSETD3; (d) Western blot for detection of cleaved caspase-3, Bax and Bcl-2 in SUNE1 cells transfected with si-circSETD3; (e) RT-qPCR for the detection of circSETD3 in SUNE1/DDP cells transfected with si-circSETD3; (f) CCK-8 for detection of the viability of SUNE1/DDP cells transfected with si-circSETD3; (g) Colony formation assay for detection of the proliferation of SUNE1/DDP cells transfected with si-circSETD3; (h) Flow cytometry detection of apoptosis of SUNE1/DDP cells transfected with si-circSETD3; (i) Western blot for detection of cleaved caspase-3, Bax, and Bcl-2 in SUNE1/DDP cells transfected with si-circSETD3. Measurement data were exposed as mean ± standard deviation (n = 3); vs the si-NC, *P < 0.05.

For the examination of the effect of circSETD3 on NPC proliferation, a significant reduction in the expression of circSETD3 in SUNE1 cells was reported in the si-circSETD3-transfected cells (Figure 2(a)), confirming that reduced circSETD3 inhibited SUNE1 cell proliferation and Bcl-2, but facilitated SUNE1 apoptosis, cleaved caspase-3, and Bax (Figure 2(b–d)). The role of circSETD3 was explored on NPC DDP resistance through the knockout of circSETD3 in SUNE1/DDP cells by transfection (Figure 2(e)). After knockdown of circSETD3, the tolerance of SUNE1/DDP cells to DDP was significantly reduced, leading to a decrease in cell viability (Figure 2(f)), and colony formation assay clarified that knockdown of circSETD3 also reduced SUNE1/DDP cell proliferation ability and Bcl-2 expression, but promoted apoptosis rate of SUNE1/DDP cells, cleaved caspase-3 and Bax expression (Figure 2(g–i)). Briefly, these observations confirmed that suppression of circSETD3 inhibits NPC proliferation and DDP resistance.

### 3.3 CircSETD3 acts as the ceRNA of miR-147a

**Figure 3.**
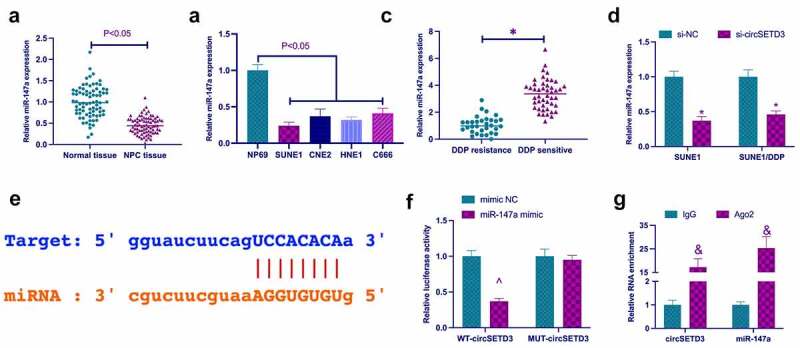
CircSETD3 acts as the sponge of miR-147a. (a) RT-qPCR detection of miR-147a in NPC tissues and adjacent normal tissues; (b) RT-qPCR detection of miR-147a in NPC cell lines (SUNE1, CNE2, HNE1, and C666) and immortalized nasopharyngeal epithelial cell lines (NP69); (c) RT-qPCR to detect the expression of miR-147a in DDP resistant and DDP sensitive patients; (d) RT-qPCR detection of miR-147a in SUNE1 and SUNE1/DDP cells transfected with si-circSETD3; (e) Bioinformatics website http://starbase.sysu.edu.cn/ query for circSETD3 and miR-147a’s potential binding sites; (f) Dual-luciferase reporting assay for detection of the targeting relationship between circSETD3 and miR-147a; (g) RIP experiment verification of the binding of circSETD3 with miR-147a. Measurement data were exposed as mean ± standard deviation (n = 3); vs the si-NC, *P < 0.05; vs the mimic NC, ^*P* < 0.05; vs the IgG, ^&^*P* < 0.05.

A previous study revealed that miR-147a performs a tumor suppressor role in non-small cell lung cancer and ovarian cancer [25,26]. However, it is unclear whether miR-147a has a similar function in NPC. In this study, miR-147a expression was examined in NPC. As shown in Figure 3(a,b), miR-147a expression in NPC patients and NPC cell lines was significantly lower than miR-147a expression in para-cancer normal tissues and NP69 cells. Subsequently, it was examined whether miR-147a was regulated by circSETD3. The results clarified that knockdown of circSETD3 significantly reduced the expression of miR-147a in SUNE1 cells and SUNE1/DDP cells (Figure 3(c)). Hence, it was hypothesized that miR-147a might be the downstream miRNA of circSETD3. The bioinformatics website http://starbase.sysu.edu.cn/ query found that circSETD3 and miR-147a had potential binding sites (Figure 3(d)). Subsequently, the targeting relationship between circSETD3 and miR-147a was further examined by dual-luciferase reporting assay and RIP assay. As revealed in Figure 3(e,f), wild-type circSETD3 significantly reduced luciferase activity in miR-147a mimic group. In addition, circSETD3 and miR-147a were significantly enriched in Ago2 compared with IgG. These results suggest that circSETD3 acts as the ceRNA of miR-147a.

### 3.4 CircSETD3 promotes NPC proliferation and DDP resistance through miR-147a

**Figure 4.**
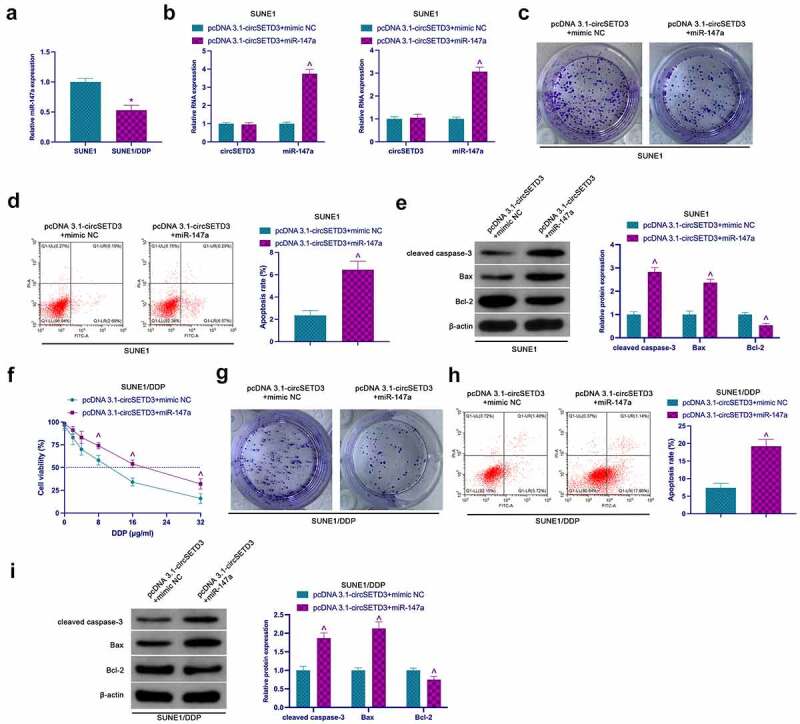
CircSETD3 accelerates NPC proliferation and DDP resistance through miR-147a. (a) RT-qPCR assessment of miR-147a in SUNE1 and SUNE1/DDP cells; (b) RT-qPCR detection of circSETD3 and miR-147a in SUNE1 and SUNE1/DDP cells transfected with pcDNA3.1-circSETD3 and miR-147a mimic; (c, g) Colony formation assay for detection of the proliferation of SUNE1 and SUNE1/DDP cells transfected with pcDNA3.1-circSETD3 and miR-147a mimic; (d, h) Flow cytometry detection of apoptosis of SUNE1 and SUNE1/DDP cells transfected with pcDNA3.1-circSETD3 and miR-147a mimic; (e, i) Western blot for detection of cleaved caspase-3, Bax and Bcl-2 in SUNE1 and SUNE1/DDP cells transfected with pcDNA3.1-circSETD3 and miR-147a mimic; (f) CCK-8 detection for the viability of SUNE1/DDP cells transfected with pcDNA3.1-circSETD3 and miR-147a mimic; Measurement data were exposed as mean ± standard deviation (n = 3); vs the SUNE1, *P < 0.05; vs the pcDNA3.1-circSETD3 + mimic NC, ^*P* < 0.05.

Next, the involvement of miR-147a in the circSETD3ʹs regulation of proliferation and resistance to DDP was investigated. According to the results shown in Figure 4(a), miR-147a expression in SUNE1/DDP cells was significantly reduced compared to SUNE1 cells. Later, pcDNA 3.1-circSETD3 and miR-147a mimic were co-transfected into SUNE1 and SUNE1/DDP cells (Figure 4(b)). The functional verification analysis confirmed that elevated circSETD3 promoted the colony-forming ability of SUNE1 cells and Bcl-2 but suppressed the apoptosis rate, cleaved caspase-3, and Bax, which was reversed by co-transfection of miR-147a mimic (Figure 4(c–e)). Similar effects were observed in SUNE1/DDP cells. Besides, the development of resistance to DDP through circSETD3 elevation was also reversed via miR-147a mimicking (Figure 4(f–i)).

### 3.5 CircSETD3 affects NPC proliferation and DDP resistance through the AKT/mTOR pathway

**Figure 5.**
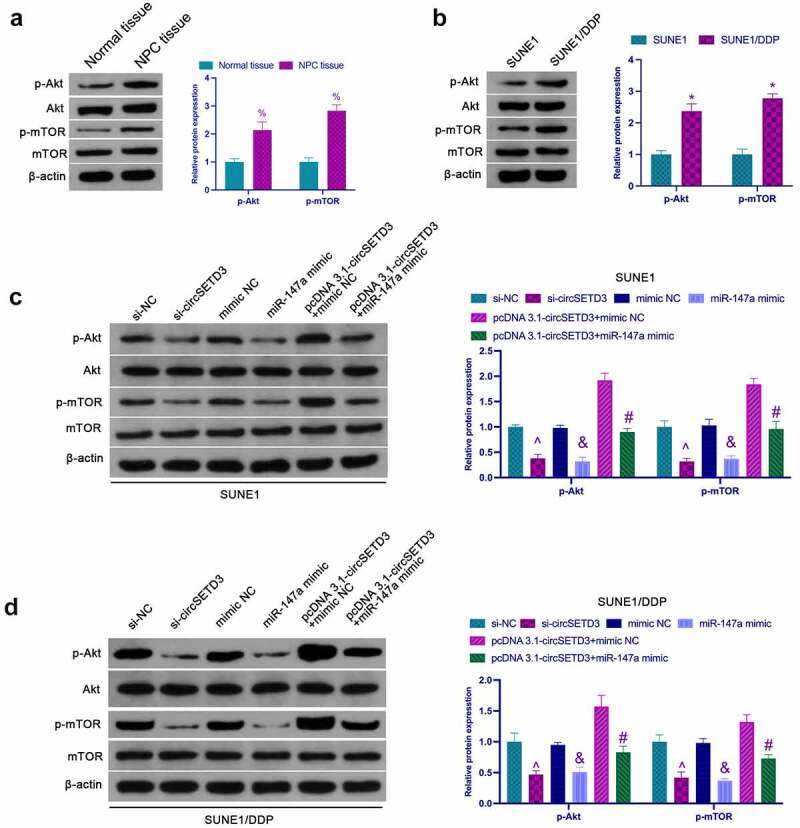
circSETD3 affects NPC proliferation and DDP resistance through the AKT/mTOR pathway. (a) Western blot detection of the expression of Akt/mTOR in NPC tissues and normal tissues adjacent to cancer; (b) Western blot detection of the activation of the AKT/mTOR pathway in SUNE1 and SUNE1/DDP cells; (c) Western blot detection of the effects of circSETD3/miR-147a on the AKT/mTOR pathway in SUNE1 cells; (d) Western blot examination of the effects of circSETD3/miR-147a on the AKT/mTOR pathway in SUNE1/DDP cells; Measurement data were exposed as mean ± standard deviation (n = 3); vs the SUNE1, *P < 0.05; vs the si-NC, ^*P* < 0.05; vs the mimic NC, ^&^*P* < 0.05; vs the pcDNA3.1-circSETD3 + mimic NC, ^#^*P* < 0.05.

Activation of the AKT/mTOR pathway is critical in cancer proliferation and chemical resistance [27]. As shown in Figure 5(a), the phosphorylation level of Akt/mTOR in NPC tissues was significantly higher than that in normal adjacent tissues. In addition, it was found that phosphorylated Akt and mTOR were significantly elevated in SUNE1/DDP cells compared to SUNE1 cells (Figure 5(b)). Next, the influence of circSETD3 in the activation of the AKT/mTOR pathway in NPV cells was examined. According to the results, knockdown of circSETD3 or up-regulation of miR-147a inhibited phosphorylated Akt and mTOR in SUNE1 cells, while circSETD3 elevation facilitated AKT/mTOR signaling, which was reversed via miR-147a enhancement (Figure 5(c)). Meanwhile, circSETD3 and miR-147a were found to have similar effects on the AKT/mTOR pathway in SUNE1/DDP cells (Figure 5(d)). Briefly, these observations confirmed that circSETD3 regulates NPC proliferation and drug resistance by activating the AKT/mTOR pathway.

### 3.6 *Effects of circSETD3 combined with DDP on tumor growth* in vivo

**Figure 6.**
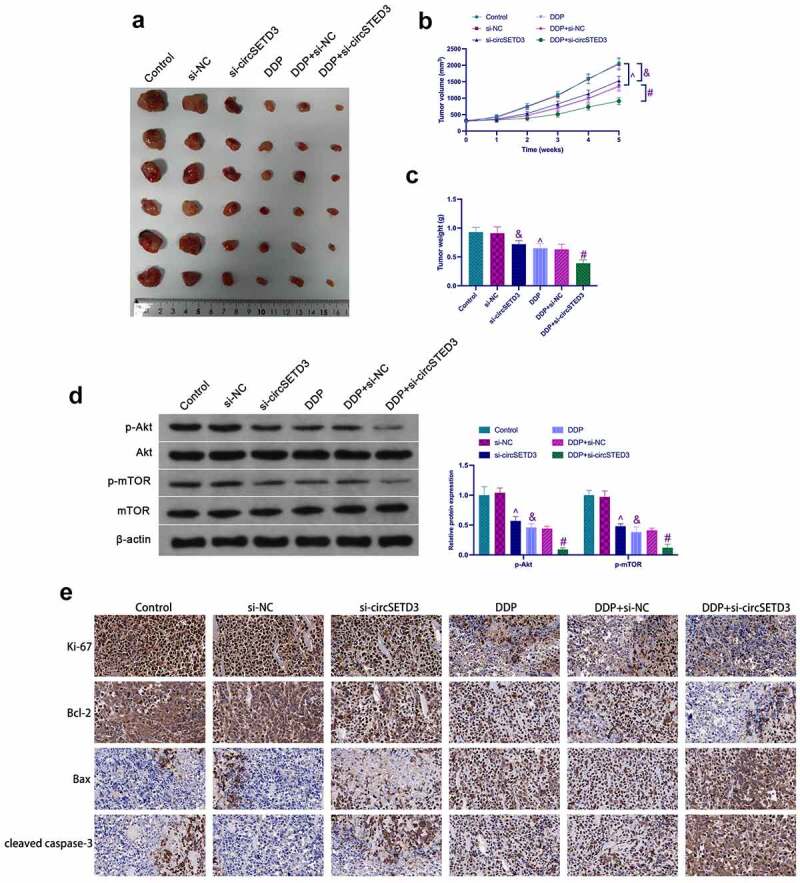
Influences of circSETD3 with DDP on tumor growth *in vivo*. (a) Representative image of tumors; (b) Tumor volume; (c) Tumor weight; (d) Western blot detection of the activation of the AKT/mTOR pathway in tumors; IHC detection of Ki67, cleaved caspase-3, Bax, and Bcl-2 expression in tumors. Measurement data were exposed as mean ± standard deviation (n = 6); vs the si-NC, ^&^*P* < 0.05; vs the si-NC + DDP, ^#^*P* < 0.05; vs the Control, ^*P* < 0.05.

To further verify the above results, *in vivo* experiments were conducted using BALB/c nude mice. The results showed that the tumor volume and weight were significantly reduced after si-circSETD3 treatment in the control and the DDP groups (Figure 6(a–c)). Moreover, si-circSETD3 also reduced phosphorylated Akt and mTOR in tumors in the control and the DDP groups (Figure 6(d)). Immunohistochemistry analysis clarified that si-circSETD3 also decreased Ki67 and Bcl-2 expression but increased cleaved caspase-3 and Bax expression in the control and the DDP groups (Figure 6(e)). These results suggest that knockdown of circSETD3 is beneficial to inhibit *in vivo* formation and DDP resistance of SUNE1 cells.

## 4 Discussion

This study confirms the importance of circSETD3 in NPC proliferation and DDP resistance. The investigation reveals that suppression of circSETD3 or up-regulation of miR-147a restrains the malignant proliferation of NPC and enhances the sensitivity of DDP. In terms of mechanism, circSETD3 acts as the ceRNA of miR-147a to promote NPC proliferation, DDP resistance, and Akt/mTOR pathway activation.

Various studies have reported the critical function of circRNAs in NPC proliferation and drug resistance. For example, Zhou M *et al*. found that circRANBP17 has an oncogenic role and increases proliferation and invasion in NPC via controlling the miR-635/RUNX2 axis [28]. CircTGFBR2 represses the proliferation and migration of NPC by serving as the sponge of miR-107, leading to a better prognosis [29]. Previous studies have confirmed that circCRIM1 increases the migration of NPC and resistance to docetaxel [30]. This study reports for the first time that circRNA has a role in the resistance of DDP in NPC. In the DDP-resistant cells, circSETD3 expression was increased.

Moreover, the knockout of circSETD3 led to elevated sensitivity of DDP-resistant cells to DDP. In addition, circSETD3 was highly expressed in NPC tissues and cell lines, consistent with previous research [15]. It is noteworthy that Xu L *et al*. reported that circSETD3 expression is inhibited in liver cancer tissues, leading to suppressed tumor growth [31]. This phenomenon suggests that circSETD3 has various effects on different types of cancers. Due to the elevation of circSETD3 in the blood, future evaluations may also include detecting serum circSETD3 levels in patients, which may consider a circulating biomarker of chemical sensitivity in NPC patients. This study further confirmed that circSETD3 promotes proliferation and DDP resistance in the SUNE1 cell line. However, the effects of circSETD3 in other NPC cell lines need more investigations. In addition, it was found that knockdown circSETD3 reduced proliferation and DDP resistance of SUNE1 and blocked activation of the Akt/mTOR pathway. Previous studies have reported that the Akt/mTOR pathway inhibition prevents the progression of NPC [19,20]. Consequently, it was speculated that the repression of NPC malignant phenotypes by circSETD3 was related to the Akt/mTOR pathway. However, future investigations using Akt/mTOR inhibitors to block the Akt/mTOR pathway are needed to check whether circSETD3 has the same effect on NPC malignant phenotypes.

Several studies have reported altering miRNA expression levels via a ceRNA of circRNAs. In the current study, the direct interaction of circSETD3 with miR-147a has been confirmed. MiR-147a has been reported to be tumor-suppressive in various cancers. For instance, miR-147a represses the growth and metastasis of NSCLC via targeting IFITM1 [32] and the progression of epithelial ovarian cancer through the down-regulation of CDK6 protein. Two studies reported a crucial role of miR-147a in radiotherapy sensitivity [33,34]. This study explored the biological function of miR-147a in NPC DDP resistance, displaying that upregulated miR-147a can reverse the increasing effect of elevated circSETD3 on NPC cell proliferation and DDP resistance. However, the downstream target genes of miR-147a in NPC proliferation and drug resistance have not been identified in this study. The specific roles of miR-147a downstream target genes in NPC proliferation and drug resistance need to be further determined in future investigations.

## 5. Conclusion

Briefly, circSETD3 was elevated in NPC patients and further strengthened after DDP treatment. The data support the role of circSETD3 as a pro-oncogene to promote NPC proliferation, DDP resistance, and Akt/mTOR pathway activation via regulating miR-147a. CircSETD3 may be a promising therapeutic target to alleviate NPC’s DDP resistance and malignant behavior.

## Highlights

1. DDP treatment elevated circSETD3 but repressed miR-147a in NPC cells;
2. Elevated circSETD3 promoted NPC proliferation and DDP resistance, but refrained apoptosis;
3. Enhancive miR-147a reversed the repressive biological behavior of NPC of strengthening circSETD3;
4. Knockdown circSETD3 or elevated miR-147a prevented Akt/mTOR phosphorylation;
5. Depressive circSETD3 elevated the DDP sensitivity of NPC cells *in vivo.*
